# Childhood IQ and adolescent health behavior

**DOI:** 10.1016/j.ssmph.2025.101887

**Published:** 2025-11-19

**Authors:** Hendrik Jürges, Rasheda Khanam

**Affiliations:** a University of Wuppertal, Gaußstr. 20, 42119, Wuppertal, Germany; b University of Southern Queensland, Toowoomba, Queensland, 4350, Australia

**Keywords:** Cognitive ability, Adolescence, Health behavior, Risk-taking, Socioeconomic inequality, Peer effects

## Abstract

This study investigates whether cognitive ability in early childhood predicts adolescent health-related and risky behaviors, independent of schooling and socioeconomic background. Using longitudinal data from the Kindergarten cohort of the Longitudinal Study of Australian Children (LSAC), we link matrix reasoning scores from ages 6 to 10 to health behaviors at age 16/17. Behaviors include substance use, unsafe driving, nutrition, dental hygiene, and sleep. To reduce dimensionality and interpret behavioral patterns, we derive two composite indices via principal component analysis: a risk-taking index and a health habit index. We find that higher early-life IQ is consistently associated with lower risk-taking and better health habits in adolescence, even after adjusting for a comprehensive set of early life covariates including non-cognitive traits, parental health behaviors, family SES, and regional disadvantages. A Gelbach decomposition shows distinct patterns of confounding: for risk-taking, the attenuation of the IQ-health behavior association is primarily explained by parental health behavior and ethnocultural background; for health habits, socioeconomic disadvantage is more salient. Peer characteristics at age 14/15 explain a substantial share of the IQ-risk-taking relationship, suggesting social environments as potential pathways. Robustness checks using the Cinelli & Hazlett sensitivity framework indicate that the IQ-health habits association is substantively and statistically robust to unobserved confounding. We interpret these findings as support for the hypothesis that early-life IQ may be an important upstream factor of health inequalities before educational differentiation occurs.

## Highlights

• Childhood IQ predicts adolescent risk-taking and health habits.
• Effects persist after adjustment for SES, personality, and family background.
• Peer characteristics mediate the IQ-risk-taking relationship.
• IQ-health habits link is robust to strong unobserved confounding.
• Supports Gottfredson's view of IQ as a health-relevant resource.

## 1 Introduction

Educational and socio-economic disparities in health and health-related behaviors are pervasive and persistent across societies (Bachman et al., 2007; Mirowsky & Ross, 2003). A large body of research has sought to identify the extent to which these disparities are causal, with a central focus on the role of education. Following Lleras-Muney's (2005) influential work, studies using compulsory schooling reforms have become the dominant approach to identifying causal effects of education. Recent evidence based on this approach suggests that the average impact of education on health is much smaller than previously assumed. For example, Clark and Royer (2013) and Begerow and Jürges (2022) found little to no causal effect, casting doubt on the strength or even existence of a direct education-health nexus.

This contrasts with persistent strong correlations between education and health in observational data. A key explanation for this discrepancy is omitted variable bias. Fuchs (1982) and Farrell and Fuchs (1982) proposed that deeper psychological traits, such as time preferences or willpower, may influence both education and health decisions. Because many health behaviors emerge already in adolescence, before formal education is completed, selection into health behaviors may be driven more by pre-existing traits than by schooling itself. In line with this, Lynch and von Hippel (2016) demonstrate that the education-health gradient reflects confounding by early-life health and cognitive factors. Additional support comes from de Walque (2010), Andersson and Maralani (2015) or Jürges and Meyer (2020), who document socio-economic gradients in adolescent health behaviors that cannot be attributed to completed schooling.

Related work highlights the role of cognitive and non-cognitive skills in shaping educational and health outcomes. Twin studies such as Wennerstad et al. (2010) found that IQ at age 18 was inversely associated with smoking 20 years later in between-twin but not in within-twin analyses, suggesting a limited role of IQ independent of shared family background. In contrast, Ciarrochi et al. (2012), find that cognitive ability assessed in Grade 7 predicts adolescent health behaviors at Grade 11, including delayed smoking onset and lower consumption of stimulant drinks, even after adjusting for conscientiousness and socioeconomic status. Cobb-Clark et al. (2014) show that non-cognitive traits such as locus of control are strongly associated with health behaviors like diet and physical activity, highlighting the importance of psychological characteristics beyond cognitive ability.

Building on this literature, we use data from the Longitudinal Study of Australian Children (LSAC) to investigate whether childhood cognitive ability (or IQ, we use both terms interchangeably) independently predicts health-related and risky behaviors during adolescence, a period in which individuals are usually still in formal education. While closely related to Ciarrochi et al. (2012), our study extends their work by using a substantially larger, nationally representative cohort, measuring cognitive ability earlier in childhood, incorporating a broader set of covariates, and exploring potential mechanisms such as peer environments (Lochner, 2011). Our study also complements other Australian data to estimate the effects of education on health behaviors. For example, Li and Powdthavee (2015) exploit variation in education due to school reforms and find limited causal effects of education on adult health habits. We add to this evidence by focusing further upstream, i.e. on cognitive ability assessed in childhood, to understand developmental predictors of adolescent health behavior.

Unlike most existing studies, we measure cognitive ability prior to or shortly after school entry, allowing us to better separate cognitive ability from formal educational exposure. Our main measure of cognitive ability is based on repeated assessments using matrix reasoning tasks from the Wechsler Intelligence Scale for Children (WISC-IV), which emphasize abstract reasoning and pattern recognition over verbal skills or acquired knowledge. Further, we study a broad set of self-reported behaviors, including legal and illegal substance use, risky driving, nutrition, preventive health behaviors, dental care, sunscreen use, and sleep patterns. These behaviors develop alongside adolescents’ educational trajectories (Jessor, 1992; Bachman et al., 2007) and reflect two underlying domains: peer-influenced risk-taking and self-regulatory health habits (Nigg, 2017; Steinberg, 2010). To reduce dimensionality and address concerns around multiple testing, we summarize them using two composite indices derived from exploratory principal component analyses: a *risk-taking index* and a *health habit index*.

To assess the relationship between early-life IQ and these indices, we proceed in several steps. First, we examine unadjusted and adjusted associations using binned scatterplots for illustration. Second, we estimate linear regression models both with and without covariates measured at baseline (age 4/5). Third, we apply Gelbach's (2016) decomposition to assess which domains of covariates attenuate the IQ coefficient. Fourth, we conduct sensitivity analyses using the approach of Cinelli and Hazlett (2020) to examine how strong unobserved confounding would need to be to fully attenuate our results. Finally, we assess whether peer characteristics at age 14/15 mediate the IQ-behavior association.

**Fig. 1:**
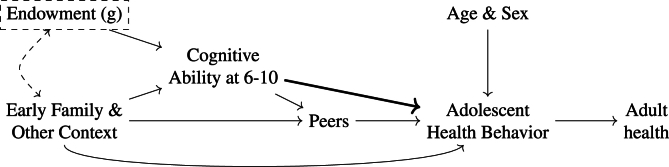
Conceptual framework. Early family/context and latent endowment (g) shape childhood cognitive ability, which may affect adolescent health behavior directly and through peers; family also exerts direct effects. Dashed elements denote latent constructs and potential correlations.

Fig. 1 summarizes our conceptual framework. Childhood cognitive ability is shaped by both social context and a latent cognitive endowment (g). Family and other context also affect adolescent health behaviors and peer environments, while peers are on the path from cognitive ability to behavior. Age and sex are included as exogenous controls. Dashed elements denote latent constructs and potential correlations (e.g., gene–environment correlation). We use this framework to motivate our adjustment set and distinguish confounding from potential pathways. The key hypothesis is that cognitive ability causally influences behavior, in line with pre-existing intelligence (g) being a fundamental cause of inequalities in adult health (Gottfredson, 2004).

Our analysis finds consistent longitudinal associations between early-life cognitive ability and adolescent behavior across the entire IQ distribution. Higher cognitive ability is associated with lower risk-taking and better health habits, even after adjusting for our rich set of covariates. Adjusted associations are smaller than unadjusted ones but remain meaningful, particularly for health habits. Peer characteristics explain a substantial portion of the association between IQ and risk-taking, suggesting that social environments during adolescence may act as a channel through which early cognitive ability affects behavior. While our design cannot establish definitive causality, our analysis suggests that cognitive ability functions as an upstream factor shaping health trajectories and contributes to persistent health inequalities.

## 2 Data and measurement

We use data from multiple waves of the Longitudinal Study of Australian Children (LSAC), a biennial panel survey. Details on this survey and its design can be found elsewhere (Mohal et al., 2023). Our analysis focuses on the Kindergarten (K) cohort, which was first surveyed at age 4/5 in 2004 and was 16/17 years old in 2016. The Infant (B) cohort surveyed at age 0/1 in 2004 turned 16/17 in 2020, when data collection was unfortunately interrupted due to Covid-19. Therefore, comparable analyses with the B cohort are not possible.

For our analyses, we link background variables measured at age 4/5 (wave 1), IQ scores averaged across ages 6 to 10 (waves 2 to 4) and behavioral outcomes at age 16/17 (wave 7). While panel attrition is substantial, it is not prohibitive: A total of 3089 children or 62 percent of the original wave 1 sample of 4983 children was retained in wave 7. Attrition in LSAC is non-random and is associated with parental socio-economic status SES. LSAC provides longitudinal weights to adjust for factors such as parental age, education, response to self-completion questionnaires, and home ownership (Usback et al., 2018). All analyses are weighted accordingly. Table A.1 in the Appendix shows unweighted and weighted descriptive statistics for comparison. Further, the wave 1 self-completion questionnaire containing important data on parental health behaviors had a unit non-response rate of 15 percent, leading to further reductions in our sample size.

### 2.1 Cognitive ability (IQ)

Cognitive ability, or intelligence, was measured at each wave. At age 4/5, LSAC employed adapted versions of two standard tests: “Who am I?” (WAI) and the Peabody Picture Vocabulary Test (PPVT). However, these tests primarily measure verbal intelligence and are arguably more susceptible to early environmental influences. WAI requires children to copy and write numbers, letters, words and sentences. It is often used to assess school readiness. PPVT is designed to assess children's receptive vocabulary, i.e., how many words they understand when they hear them.

Our view of intelligence is that it partly reflects pre-existing cognitive ability independent of formal education. Instead of WAI and PPVT, we therefore use the Matrix Reasoning (MR) test from the Wechsler Intelligence Scale for Children (WISC-IV), administered at ages 6/7, 8/9, and 10/11. The MR test assesses non-verbal intelligence by asking children to complete a set of increasingly complex diagrams. The test is designed to measure pattern recognition, abstract reasoning, and nonverbal problem-solving independent of prior knowledge, which is closer to the concept of pre-existing cognitive ability not affected by formal and informal education itself.

LSAC provides both raw scores (number of correct responses) and scaled scores based on age norms from the WISC-IV manual (mean = 10, SD = 3). Following Cahan (2000), we centered raw test scores (non-parametrically) on group averages defined by age in months and half school grades, thereby implicitly adjusting for age and grade at the time of measurement. Additionally, we centered on sex averages to counteract sex differences due to developmental differences in early childhood or differences in test-taking behavior (raw test scores were statistically significantly lower for boys than girls). To minimize measurement error, we computed the sum of the test scores across the three waves, standardized this sum and used the resulting composite score in our analyses.

### 2.2 Risk-taking and health habits

Risky behavior and health habits were measured primarily through self-reports using computer-assisted self-interviewing (CASI) to reduce interviewer bias and the tendency for socially desirable responses (Brener et al., 2003). In this paper, we use 14 different binary measures of behavior:

• Use of legal and illegal substances: smoking, drinking alcohol, trying marijuana, trying other drugs
• Risky driving behaviors: not wearing seatbelt or helmet, driving under the influence of alcohol (also as passenger), driving 25 km/h or more above speed limit
• Nutrition: consumption of high sugar drinks, high fat foods, fruits and vegetables
• Prevention and self-care: frequency of brushing teeth, frequency of dentist visits, using sunscreen
• Sleep patterns: Sleeping less than 7 or more than 9 h on a typical school day

Further details including the exact wording of the survey questions can be found in Appendix B.1. Following reviewer feedback, we excluded sexual activity from the analysis to avoid conflating normative adolescent development with health risk behaviors. Further, regarding driving behavior, we note that in LSAC, 70 % of 16/17 year olds report having a learner driver's permit (i.e. L's, L1's, L2's); 12 % report having a provisional driver's license (i.e. P's, P1's, P2's); and 18 % do not hold any driver's license.

Table 1 summarizes the prevalence of (un-)healthy and risky behaviors at age 16/17. Approximately 10 percent of the cohort engaged in risky behaviors such as smoking regularly, trying marijuana, or speeding, whereas drinking alcohol and bad habits such as neglecting tooth hygiene or eating unhealthy food are more common (around 30 percent). However, since the reference periods for different types of behavior are different, care must be taken when directly comparing the frequency across measures.

**Table 1:** Percentage of adolescents engaging in risky or unhealthy behavior age 16/17. Component loadings derived from PCA with varimax rotation (N = 2253).

Variable	Reference period	Proportion	Loadings 1	Loadings 2
Risk-Taking				
Smoking last week	Last 7 days	0.08	0.70	0.05
Alcohol last week	Last 7 days	0.29	0.51	−0.08
Ever tried marijuana	Ever	0.09	0.72	0.08
Ever tried other drugs	Ever	0.10	0.65	0.08
No seatbelt/helmet	Last 10 drives	0.10	0.41	0.07
Speeding>25 km/h	Last 10 drives	0.08	0.46	−0.00
Drunk driving	Last 10 drives	0.11	0.59	0.06

Health Habits				
Brushed teeth <2 times	Yesterday	0.35	0.01	0.65
Dentist visit >1yr ago	Last years	0.30	0.02	0.46
Sleep <7 or >9hrs	Usual school day	0.24	0.01	0.24
Not using sunscreen	When outside during summer	0.16	0.11	0.53
High fat food/day	Yesterday	0.29	0.05	0.43
High sugar drinks/day	Yesterday	0.37	0.06	0.47
Fruit and vegs/day	Yesterday	0.35	−0.02	0.44

Proportion of explained variance			0.60	0.40

Based on exploratory Principal Component Analysis (PCA), we computed two composite indices as unweighted, standardized sum scores (with mean 0 and SD 1) of the binary indicators corresponding to the high-loading items. This helps to avoid issues of multiple testing, reduces the amount and complexity of our results, and allows for easier interpretation of our findings compared to analyzing 14 outcomes simultaneously. The two scores will be our analytical focus. The first score is labeled “risk-taking index” and includes the 7 items related to substance use, and careless driving, i.e. often impulsive or antisocial behavior, possibly influenced by peers, sensation-seeking, or reduced adult supervision. The second index is labeled “health habit index” and includes the 7 items related to nutrition, prevention, and sleep, i.e. preventive health behaviors and self-care, and self-regulation. Table 1 shows the results of a residualized PCA (after accounting for age at measurement) with two principal components. Varimax rotation was applied to aid interpretability. We accounted for age as a common factor because especially risk-taking varies naturally with the age at which it is measured. PC loadings regarding the risk-taking index are moderate to strong, PC loadings regarding the health habits index are predominantly moderate. Also see Appendix Figure A.2 for a graphical representation.

### 2.3 Observed covariates

We leverage the strength of LSAC and rely on controlling for a wide range of plausible covariates. These covariates are potentially simultaneously correlated with IQ in childhood and health-related behavior in adolescence. We also adjust for further characteristics that affect the outcome but not the treatment may be adjusted for as well in order to increase the precision of our estimates. On the other hand, we avoid controlling for characteristics that are likely affected by IQ itself and are hence on the path from IQ to health-related behavior (i.e., mediators such as peer characteristics). We distinguish between sets of covariates belonging to conceptually separate domains.:

1. Age & Sex: sex and age at IQ test taking are adjusted for implicitly by standardizing the test score. Further, we also corrected the risk-taking and health habits index for the exact age at which the questions were asked. Nevertheless, we include sex and age and an interaction term to account for any remaining sex specific differences in behavior and sex differences in age trajectories.
2. Ethno-cultural background: measured by mother's world region of birth according to the Standard Australian Classification of Countries (SACC) 2011. We include this variable mainly to capture ethno-cultural differences in behaviours such as substance use.
3. SDQ: non-cognitive skills at age 4/5 as measured by the strengths and difficulties questionnaire, administered to parents (Goodman, 1997). The four subscales emotional problems, conduct problems, inattention/hyperactivity and peer problems are used separately.
4. Parental Behavior: parental and other household members' behavior when the child was 4/5 years old: mother's and father's current drinking and smoking; number of household members who smoke inside the household; presence of older siblings, mother's diet (fruit and vegetable consumption), parental engagement measured by a home activities index (e.g., reading to child, drawing picture with child, etc.), and an out-of-home activities index household activities with child (e.g., visiting museums, sporting events, etc.).
5. Family SES: Number of children's books at home, mother's and father's highest education level combined; mother's English proficiency, mother's and father's age; mother's labor force status; home ownership; how the family is getting on financially; financial hardship scale; Z-score for socioeconomic position among all families.
6. Regional/School SES: remoteness of statistical local area (SLA); local area index of relative socio-economic advantage and disadvantage (SEIFA); school community socio-educational advantage (ICSEA) and school average grade 5 NAPLAN score when child was in grade 5 (age 10/11).
7. Peers: peer characteristics and behavior when the study child was 14/15 years old were measured by 20 self-reported items such as “They work hard at school”, “They smoke cigarettes”, etc. The items are summarized in three sum scores: “Peer group positive orientation toward academic achievement”, “Peer group moral behavior”. “Peer risk-taking”. The full list of items and their allocation to one of the three indices can be found in Appendix B.2. Peers are arguably the most important mediating factor when analyzing teenage behavior, we use those variables to gauge their role in additional analyses, although we do not claim to conduct a full mediation analysis.

In single mother households, fathers' variables were replaced by the median or mode of valid responses and additionally flagged by a “missing” dummy variable that was also included in the analyses below. Due to our definition of mother and father, which we take from LSAC, single-father households are excluded from the analysis. In same-sex couples, which were exclusively female, the mother is whoever identified as biological mother, and the father's information is coded as missing.

To facilitate interpretation and comparability across predictors, all continuous variables were standardized by dividing by two times their standard deviation**,** following the approach proposed by Gelman (2008). This scaling allows the resulting coefficients to be interpreted as the expected change in the outcome associated with a shift from one standard deviation below the mean to one standard deviation above the mean, making them directly comparable to the coefficients of untransformed binary variables. Summary statistics for all covariates are listed in Appendix Table A.1.

## 3 Analytical strategy

Our main goal is to examine whether LSAC data support the claim that intelligence acts as a fundamental cause of health inequalities (Gottfredson, 2004). Specifically, we assess whether early-life IQ is associated with adolescent risky and health-related behaviors in a manner that is plausibly causal, conditional on extensive adjustment for observed covariates and sensitivity checks. One important characteristic of our approach is the time structure, measuring intelligence in childhood and health habits and risky behavior in adolescence. Hence, reverse causality, a possibility that plagues analyses in cross-sectional data can be ruled out as an explanation for the link between IQ and health behaviors. Our main analysis proceeds in several steps.

**Binned scatterplots:** Binned scatter plots provide a non-parametric visualization of the bivariate relationships and serve as an intuitive diagnostic tool prior to adjustment (Cattaneo et al., 2024a, Cattaneo et al., 2024b). We use binned scatterplots to illustrate the unadjusted relationship between early cognitive ability and each individual adolescent behavior as well as the “risk-taking” and “health habit” sum scores. That is, we divide the matrix reasoning sum scores into quantiles, compute the average cognitive ability score as well as the percentages of the outcome variables within each quantile and plot those means against each other in a scatterplot. The numbers of quantiles vary between outcomes and are determined by the data itself. Moreover, we add linear (global) fits together with 95 % confidence bands to the binned scatter plot. Depending on the type of outcome (binary for individual behavior items, continuous for indices) we use logit or OLS individual-level regression models to compute fitted values and confidence intervals. For the risk-taking and health habit scores, we also estimated covariate-adjusted binscatter plots (accounting for all of our covariates) via semiparametric partially linear regression (Cattaneo et al., 2024b). This helps to detect possible functional form issues in the IQ-behavior relationship in the fully controlled regression models.

**Multiple linear regression:** The next step is to estimate two linear regression models per outcome, one base regression (without adjustment for any covariates) and a full regression (with adjustment for all covariates). All models use longitudinal weights provided by LSAC and report standard errors clustered at the primary sampling unit (PSU) level. Statistical significance is assessed at the p < 0.05 level unless otherwise noted. Our coefficients of interest are the regression coefficients of cognitive ability in the full regressions. Under the assumption of homogeneous effects (i.e., linear relationships constant across sub-groups) and no unobserved confounders they identify the average treatment effect of a two standard deviation change in cognitive ability on risk-taking and health habits. Full regression results, including all control variables, are shown in Appendix Table A.2.

**Decomposition analysis:** In order to learn which set of covariates changes the IQ coefficient most, we refrain from sequentially adding different sets of covariates. Rather we use Gelbach's (2014, 2016) decomposition to attribute the total change in the IQ coefficient between the unadjusted and fully adjusted models to distinct blocks of covariates. This approach avoids order dependence and provides a statistically grounded estimate of each domain's contribution to attenuation.

**Sensitivity analysis:** An important question is whether there are any relevant unmeasured confounders introducing bias in the estimated IQ-health behavior relationship. Of course, this is inherently untestable. However, we follow the approach suggested by Cinelli and Hazlett (2020) in order to quantify the plausibility of this assumption in the linear model. That is, we use the robustness value (RV) to assess how strongly a hypothetical unobserved confounder must be correlated with the treatment and the outcome in order to push the coefficient of interest to zero (or to make it statistically insignificant at the 5 % level). Further, we compare a hypothetical unobserved confounder with the strength of actual (observed) sets of covariates in our full model. This provides a transparent benchmark for evaluating the credibility of the unconfoundedness assumption.

Note that the Gelbach and the Cinelli & Hazlett analyses are structurally related. While the Gelbach method decomposes realized shifts in coefficient estimates using observed data, Cinelli & Hazlett extend this logic to hypothetical confounders, allowing for sensitivity analysis under varying assumptions about confounding strength. Both methods rely on the algebra of omitted variable bias, linking the influence of a variable to its explanatory power for treatment and outcome.

**Peers as mediators:** As discussed, peer characteristics are arguably an important mediator on a causal path from IQ to behavior, as more intelligent children are likely to choose more intelligent and arguably better behaving peers. Peer quality might also vary with family, school, and regional environments. To explore whether peers serve as a pathway linking IQ to adolescent behavior, we extend the Gelbach decomposition to include peer characteristics measured at age 14/15. Our measures comprise adolescents’ reported attitudes toward school, morality, and substance use – traits that are plausibly shaped by earlier cognitive development and may in turn influence behavioral outcomes. While these variables are measured after IQ and before the outcomes, and thus plausibly lie on the causal pathway, we emphasize that this analysis is exploratory and not a formal mediation model.

## 4 Results

### 4.1 Binscatter plots

**Fig. 2:**
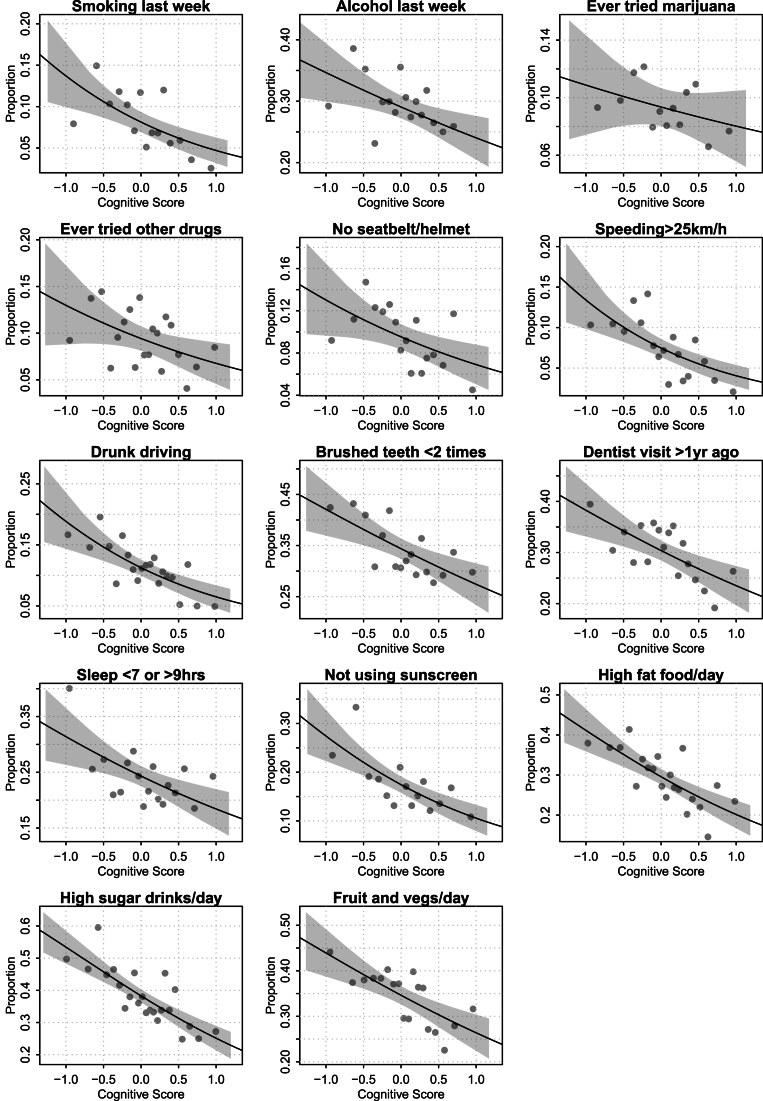
Binned scatterplots of scaled test scores at age 6 to 10 and individual behaviors at age 16/17. Confidence bands of individual-level logistic regressions based on robust standard errors. All estimates use longitudinal weights to account for selective drop-out between measurements. Estimated with R-package binsreg (Cattaneo et al., 2024b).

**Fig. 3:**
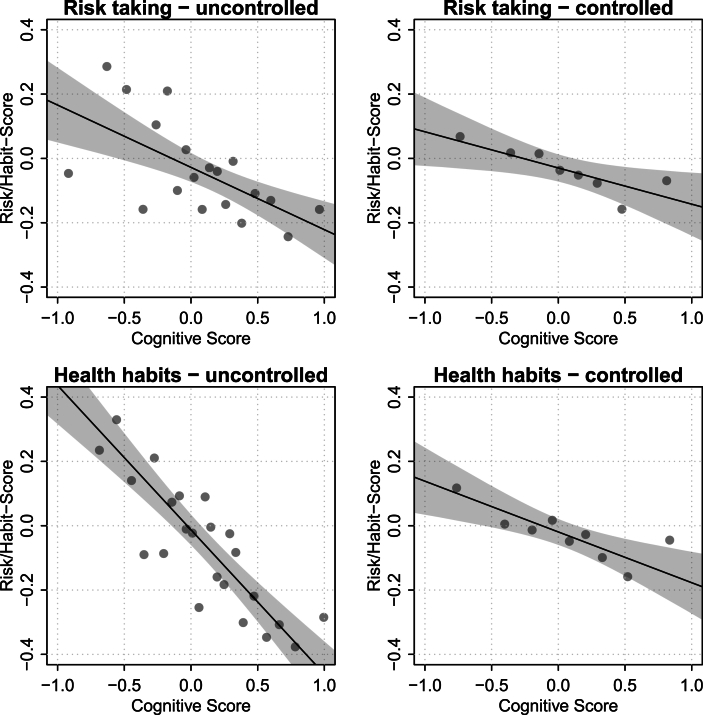
Binned scatterplots of scaled test scores at age 6 to 10 and risk-taking and health habits at age 16/17. Controlled relationships include all sets covariates. Confidence bands of individual-level linear regression based on robust standard errors. All estimates use longitudinal weights to account for selective drop-out between measurements. Estimated with R-package binsreg (Cattaneo et al., 2024b).

Binscatter plots showing unadjusted associations between childhood cognitive ability and adolescent risky and health-related behaviors are presented in Fig. 2, Fig. 3. Across all behaviors, higher childhood IQ is associated with a lower likelihood of engaging in risk-taking activities during adolescence, such as smoking, substance use, and speeding. Similarly, adolescents with higher childhood IQ are less likely to report unhealthy habits like poor diet, infrequent dental care, or lack of sunscreen use. These associations are captured in the composite indices (Fig. 3), both of which display strong negative linear trends with IQ, particularly the health habits index. The observed relationships appear graded, with behavioral variation evident across the entire IQ distribution, not just at the extremes. Controlling for our wide range of covariates, the adjusted scatterplots show a much-attenuated relationship. In the following analysis we quantify and decompose these shifts.

### 4.2 Regression and decomposition analysis

Table 2 presents the results from the linear regression models (Panel A) and Gelbach decomposition analysis (Panel B). In the unadjusted models, a two standard deviation increase in cognitive ability is associated with a 0.185 SD reduction in the risk-taking index and a 0.428 SD reduction in the health habits index. After adjusting for all observed covariates, these partial associations attenuate to −0.107 and −0.151, respectively but remain statistically significant (p = 0.012 and p < 0.001). While their magnitudes are reduced by roughly one half, the partial associations remain meaningful, particularly in light of the potential for cumulative behavioral impacts on long-term health outcomes. To place the adjusted IQ coefficients in context, we compare them with the coefficients of other variables (see Table A.2 for detailed results). For example, the relationship between risk-taking and IQ is a bit larger than with having no older sibling (0.077), slightly smaller than with peer school achievement as indexed by standardized year 5 NAPLAN scores (−0.165), and 40 percent that of maternal smoking when the child was 4 years old (0.256). The relationship of IQ with health habits is similar to having no older sibling and school average NAPLAN scores and having a smoking mother. Thus, across both outcomes, IQ shows partial associations that are similar to those linked to important family background characteristics and school performance.

**Table 2:** Linear regression and Gelbach Decomposition.

	Dependent variable: risk-taking			Dependent variable: health habits		
Panel A. Regression results						
	β	SE	ModelR-squared	β	SE	ModelR-squared
Unadjusted model (β)	−0.185	0.043	0.011	−0.428	0.048	0.047
Adjusted model (β)	−0.107	0.043	0.095	−0.151	0.044	0.269

Panel B: Gelbach Decomposition						
	Δβ	SE	% of Δβ	Δβ	SE	% of Δβ
Total shift (Δβ)	−0.077	0.028	100 %	−0.278	0.033	100 %

Age & sex	0.001	0.003	−1.2 %	0.003	0.010	−0.1 %
Ethno-cultural background	−0.021	0.010	26.6 %	0.001	0.011	−0.3 %
Strengths & difficulties	0.001	0.010	−1.5 %	−0.043	0.012	15.5 %
Family behavior	−0.042	0.019	53.7 %	−0.075	0.017	26.9 %
Family SES	−0.006	0.020	7.2 %	−0.086	0.023	31.0 %
Regional/school SES	−0.012	0.016	15.2 %	−0.078	0.016	28.0 %

Notes: N = 2253. Standard errors are clustered on primary sampling units. Panel A: Adjusted model includes the full set of covariates as listed in Section 2.3 (except peers). Panel B: each row shows the contribution of a covariate block to the explained change in the IQ coefficient when moving from a base to a full model. ‘Total shift (Δβ)’ reflects the total change in the IQ coefficient. Percentages indicate each variable block's share of the total shift. Negative percentages imply suppression. Gelbach decomposition estimated in Stata using b1x2 (Gelbach, 2014).

Using the Gelbach decomposition, we now quantify how much of the difference between the controlled and uncontrolled models is statistically explained by our six pre-defined groups of covariates: age and sex, ethno-cultural background, child internalizing and externalizing behavior (SDQ), parental behavior, family SES, and regional/school-level SES.

Panel B of Table 2 shows that for risk-taking, the attenuation of the IQ coefficient is driven primarily by family behavior, which explains 53.7 % of the total reduction. This is followed by contributions from ethno-cultural background (26.6 %), regional/school SES (15.2 %), and to a lesser extent, family SES (7.2 %). These results show that behavioral patterns in the home including ethno-cultural background are the main covariates driving much of the observed bivariate association of childhood IQ and risk-taking. Even when considered together, family and regional/school SES account for less than half the statistical importance of family behavior.

For health habits, the reduction in the IQ coefficient is explained by a more evenly distributed set of confounding factors. Family SES contributes the largest share at 31.0 %, followed closely by regional SES (28.0 %), family behavior (26.9 %), and SDQ (15.5 %). Compared to risk-taking, the SES variables thus play a larger role in explaining the association of childhood IQ effect with health habits, suggesting that the association is more sensitive to background economic conditions and social disadvantage.

Taken together, the decomposition reveals distinct patterns of confounding for the two outcomes: for risk-taking, home environment and cultural background dominate, whereas for health habits, material disadvantage and socioeconomic context appear more influential.

### 4.3 Sensitivity analysis

The identifying assumption of no unobserved confounders is key to interpret the controlled models’ coefficients as causal effects. We have seen that including our wide range of covariates has dampened the association between childhood IQ and adolescent outcomes by about one half (risk-taking) or two thirds (health habits). These reductions leave open the possibility that (yet unobserved and uncontrolled) confounders would further attenuate our estimated effects, possibly to zero. We now quantify the strength of a hypothetical confounder that would be necessary to bring the coefficient of interest to zero or render it statistically insignificant, following the approach suggested in Cinelli and Hazlett (2020).

Panel A of Table 3 reports robustness values, which quantify this strength in terms of partial R-squared. The robustness value to reduce the IQ effect to zero is 0.053 for risk-taking and 0.077 for health habits. This means a confounder would need to account for roughly 5 % or 8 % of the residual variation in both treatment and outcome to eliminate the association completely. The thresholds for rendering the effect statistically insignificant at the 5 % level are lower (0.008 and 0.037, respectively), but at least in the case of health habits still nontrivial. These values suggest that the observed associations are reasonably robust to moderate omitted variable bias.

**Table 3:** Sensitivity analysis of IQ effects on adolescent outcomes.

	Risk-taking				Health habits			
Panel A: Robustness Values								
Partial R^2^ of treatment with outcome (R^2^_Y ∼ D|X_)	0.003				0.006			
Robustness value (q = 1)	0.053				0.077			
Robustness value (q = 1, α = 5 %)	0.012				0.037			

Panel B: Bounds Based on Benchmark Covariates								
Benchmark Variables	Adj. Est.	Adj. SE	R^2^_D ∼ Z|X_	R^2^_Y ∼ Z|D,X_	Adj. Est.	Adj. SE	R^2^_D ∼ Z|X_	R^2^_Y ∼ Z|D,X_
Age & sex	−0.105	0.038	0.000	0.002	−0.141	0.039	0.000	0.065
Ethno-cultural background	−0.091	0.038	0.008	0.009	−0.141	0.038	0.008	0.003
Strengths & difficulties	−0.088	0.037	0.023	0.005	−0.118	0.039	0.023	0.013
Family Behavior	−0.054	0.038	0.023	0.035	−0.097	0.041	0.023	0.036
Family SES	−0.073	0.038	0.021	0.016	−0.103	0.039	0.021	0.031
Regional/school SES	−0.088	0.038	0.015	0.007	−0.112	0.039	0.015	0.028

Adjusted model (β)	−0.107	0.043			−0.151	0.044		

Notes: N = 2253. Panel A reports robustness values based on the Cinelli and Hazlett (2020) method. The robustness value indicates the minimum strength of an unobserved confounder, in terms of partial R^2^ with both treatment and outcome, required to reduce the estimated effect by 100 % (q = 1), i.e. to zero, or render it statistically insignificant from zero at the 5 % level. Panel B reports bounds under hypothetical confounders calibrated to match the strength of sets of observed covariates. Partial R^2^ values are shown for each benchmark variable's association with the treatment and outcome, respectively. R^2^_D ∼ Z|X_: share of residual variance of the treatment D (IQ) explained by the omitted variable Z, after accounting for the remaining covariates X (so that Z is as strong as the benchmark); R^2^_Y ∼ Z|D,X:_ share of residual variance of the outcome Y (Risk behavior) explained by the omitted variable Z, after accounting for the remaining covariates X and the treatment D (so that Z is as strong as the benchmark). Standard errors estimated by block (PSU) bootstrap with 1000 repetitions to account for sample design. All estimations used R-package sensemakr (Cinelli et al., 2024).

Partial R-squared values in microdata are typically small, anyway, so it is helpful to benchmark robustness thresholds against the explanatory power of actual observed covariates. Our bounds analysis helps to develop an understanding whether 5 % or 8 % of the residual variation needed to attenuate the coefficient of interest completely could plausibly attained by a hypothetical confounder. This makes the robustness of our findings transparent and eventually can be evaluated by the reader based on their own judgement. Building on this approach, Panel B of Table 3 explores the sensitivity of the IQ-risk-taking relationship under hypothetical confounding calibrated to match the strength of our six sets of observed covariates. If such confounding was exactly as strong as the confounding of SDQ, family behavior, or SES, respectively, the adjusted coefficients would shrink but remain consistently negative. In some cases, however, they would fall below the 5 % threshold for statistical significance. For example, under the assumption that a confounder was as strong as family behavior, the adjusted estimate would fall to −0.054 (SE = 0.038). Since family behavior accounts for over 50 % of the IQ-health behavior correlation, we interpret this as the IQ-risk-taking association being directionally robust but statistically fragile to quite strong unobserved confounding. Whether the loss in statistical significance is just due to lack of statistical power or due to a lack of strength of the relationship cannot be answered without further data.

In contrast, the IQ-health habits association is statistically and substantively robust across all benchmark scenarios. Under confounding as strong as SDQ or family SES, the adjusted effect remains statistically significant and sizable—for instance, −0.118 (SE = 0.039) with a confounder as strong as SDQ and −0.103 (SE = 0.039) with a confounder as strong as family SES. Even assuming confounding strength equivalent to that of family behavior, the coefficient remains statistically significant (−0.097, SE = 0.041). These results demonstrate that the IQ-health habits relationship is unlikely to be explained away by unobserved confounders comparable in strength to groups of key observed covariates, strengthening confidence in the robustness of this association.

### 4.4 The role of peers

We focus on peer characteristics as a potential pathway linking cognitive ability to adolescent health behavior, given the well-documented influence of peers during this life stage. LSAC provides particularly rich and relevant data on peer environments, which allow us to explore this mechanism in greater depth than most comparable studies. Table 4 presents the results of the Gelbach decomposition accounting for peers. Including peer characteristics reduces the sample size (from 2253 to 2132) due to missing data. This may slightly reduce coefficient precision but also affect comparability with our main results. Therefore, we have re-estimated the uncontrolled coefficients and the controlled coefficients (but not including peers) with the reduced sample.

**Table 4:** Regression and Gelbach decomposition including peer effects.

	Dependent variable: risk-taking			Dependent variable: health habits		
Panel A. Regression results						
	β	SE	R-squared	Β	SE	R-squared
Unadjusted model (β)	−0.167	0.044	0.009	−0.393	0.047	0.039
Adjusted model (β)	−0.084	0.041	0.252	−0.133	0.045	0.268

Panel B: Gelbach Decomposition						
	Δβ	SE	% of Δβ	Δβ	SE	% of Δβ
Total shift (Δβ)	−0.083	0.029	100.0 %	−0.260	0.033	100.0 %

Age & sex	0.002	0.002	−1.9 %	0.003	0.010	−0.1 %
Ethno-cultural background	−0.009	0.008	0.8 %	0.006	0.010	−0.3 %
Strengths & difficulties	0.011	0.008	−13.7 %	−0.038	0.011	14.6 %
Family behavior	−0.029	0.016	35.4 %	−0.051	0.016	19.5 %
Family SES	0.003	0.017	−03.4 %	−0.083	0.021	32.0 %
Regional/school SES	−0.008	0.014	9.2 %	−0.067	0.015	25.9 %
Peer characteristics	−0.053	0.016	63.7 %	−0.030	0.009	11.4 %

Notes: N = 2132. Standard errors are clustered on primary sampling units. Panel A: Adjusted model includes the full set of covariates as listed in Section 2.3. Panel B: Each row shows the contribution of a covariate block to the explained change in the IQ coefficient when moving from a base to a full model. ‘Total shift (Δβ)’ reflects the total change in the IQ coefficient. Percentages indicate each variable block's share of the total shift. Negative percentages imply suppression. Gelbach decomposition estimated in Stata using b1x2 (Gelbach, 2014).

Despite this limitation, the patterns of decomposition remain informative, especially in highlighting the role peer environments may play as potential pathways through which early cognitive ability relates to adolescent risk-taking behavior. Peer characteristics explain only slightly less than two thirds (63.7 %) of the total shift in the IQ coefficient. This supports the interpretation that peer environments serve as important mechanisms through which early cognitive ability affects adolescent risk-taking behavior, either through peer selection, social learning, or susceptibility to peer norms. Remarkably, the addition of peer characteristics reallocates explanatory power rather than merely adding to it. Peer and family behavior together account for nearly 100 % of the IQ coefficient shift.

In contrast, for health habits, peer variables explain only 11.4 % of the IQ effect shift, which is consistent with previous results emphasizing the roles of family SES (32.0 %), regional/school SES (25.9 %), and family behavior (19.5 %) as the primary explanatory factors. The relatively small contribution of peer influences suggests that while peers may play a role in shaping some health-related behaviors, structural and familial conditions are more central to the IQ-health habits relationship. The contrast across outcomes underscores that mediation pathways differ in both strength and character.

Our findings suggest that peers may play an important intervening role in shaping how early cognitive ability translates into adolescent behavioral outcomes. Specifically, youth with higher IQs may be more likely to associate with peers who value school, reject risky behaviors, and reinforce positive norms – mechanisms that could help explain part of the protective association observed. While the peer variables cannot be treated as formal mediators without stronger causal identification, the descriptive contribution of peer characteristics supports the idea that social context in adolescence may partially carry forward the influence of earlier cognitive ability.

## 5 Discussion

This study demonstrates a consistent and graded association between early-life cognitive ability and health-related behaviors during adolescence. Although our design does not allow for definitive causal claims, the observed patterns are robust to extensive covariate adjustment and sensitivity analyses, and they are consistent with childhood IQ functioning as an upstream factor influencing adolescent behavior. In particular, we find that higher IQ at ages 6 to 10 is associated with lower risk-taking and better health habits at age 16/17, with associations evident across the full IQ distribution. This pattern parallels the well-established socio-economic gradient in health, where better health outcomes are linked to higher socio-economic status. The size of our estimated adjusted associations (11 %–15 % of a standard deviation per two standard deviation difference in IQ) may seem small. We argue, however, that they are meaningful not only because they comparable in size to the coefficients of important covariates, statistically significant at the 5 % level and robust to sensitivity checks, but especially because the underlying behaviors they capture (e.g., smoking, diet, self-care) typically accumulate and compound across the life course.

Our study improves on prior research in several ways. First, we leverage longitudinal IQ measurements before or soon after school entry, minimizing the conflation of intelligence with educational exposure. Second, the two composite indices we develop, risk-taking and health habits, capture distinct behavioral domains and allow us to examine how different dimensions of adolescent behavior relate to early cognitive ability.

Decomposition analyses reveal that these two domains are shaped by different underlying factors. For risk-taking, the attenuation of the IQ association is largely explained by family health behaviors and ethno-cultural background, consistent with social modeling. In contrast, the IQ-health habits relationship is more strongly confounded by socioeconomic factors such as family SES, regional/school disadvantage – factors linked to structural support. This divergence suggests that while both behavior types are cognitively influenced, they are embedded in different psychosocial and material contexts.

Peer environments appear particularly important in shaping risk-taking behavior. When peer characteristics, measured at age 14/15, are included in the decomposition, they alone account for two thirds of the IQ-risk-taking association. This is consistent with the idea that higher cognitive ability is linked to peer selection processes that may in turn shape adolescent behavior. In contrast, peer characteristics play a much smaller role in shaping health habits, suggesting that these are less susceptible to adolescent social influence and more rooted in early family and environmental conditions. While we refrain from interpreting these peer variables as formal mediators, the results highlight that part of the observed association between IQ and behavior operates through social pathways. This aligns with broader models of adolescent development in which cognition shapes not only individual decision-making, but also the selection into and response to social environments.

Our findings are compatible with the idea that cognitive ability, like socioeconomic status or health literacy, may function as a flexible resource that enables individuals to avoid health risks and adopt protective behaviors across a range of contexts. Even prior to the completion of schooling, more cognitively able youth may be better equipped to process information, anticipate long-term consequences, and navigate peer influence – thus contributing to persistent inequalities in health trajectories. At the same time, our findings should not be interpreted as support for a deterministic view of intelligence. As Mirowsky and Ross (2003) argue, intelligence itself is shaped by education and developmental context, not merely inherited. While we use early IQ as a predictor, we do not claim that it is immutable or immune to environmental inputs. Our focus is on how early cognitive advantage may initiate behavioral trajectories.

Several limitations must be acknowledged. First, our behavioral outcomes rely on self-reported data. Their validity may be affected by cognitive or situational factors (Brener et al., 2003), with individuals under- or overreporting unhealthy or deviant behaviors. In interpreting our findings, it is important to consider the possibility that the link between intelligence and adolescent health behaviors may be influenced by systematic misreporting, particularly social desirability bias. However, while there is substantial literature on social desirability bias in adolescent health risk behaviors, the specific relationship between cognitive ability/intelligence (or more broadly SES/education) and the propensity for social desirability bias appears understudied.

Extant studies focus on demographic factors, possibly correlated with IQ. For example, Edoka (2017) compared adolescents’ self-reported smoking and cotinine measurements (serving as gold standard). The SES indicator in this study was log parental income. Adolescents from wealthier households were more likely to under-report and less likely to over-report smoking, however, the differences were not statistically significant. Crutzen and Göritz (2010) investigated the relation between scoring high on a social desirability scale and self-reported health risk behaviors in web-based research. They found no meaningful associations between social desirability and self-reported health risk behaviors, and education generally did not moderate the relationship between social desirability and self-reported health risk behaviors.

While the evidence for differential reporting bias by cognitive ability is weak, reporting bias as such likely attenuates the estimated effects of cognitive ability and health behaviors. We note, however, that our models explicitly controlled for parental education and socioeconomic background, which shape both actual behaviors and adolescents’ willingness to disclose them. Further, we control for child psychosocial adjustment (SDQ), which may capture variation in self-presentation tendencies. These steps strengthen confidence that the estimated association between intelligence and health behaviors is not simply an artifact of differential reporting.

In addition, while sensitivity analysis following Cinelli and Hazlett (2020) suggest that our results are moderately robust to unmeasured confounding, we cannot fully rule out the possibility of important confounders. Although we strive to estimate the effect of “innate” cognitive ability by adjusting for a wide range of environmental factors, the remaining IQ measure may still contain social and cultural influences, and separation of pre-existing and education-caused IQ (Mirowsky & Ross, 2003) is incomplete.

Our findings have several implications for public health policy. Given the links between cognitive ability, risk-taking and health habits, early interventions aimed at improving cognitive ability could have long-lasting effects on health outcomes. As behaviors established in adolescence often persist in adulthood, the adolescent period represents a critical window for supporting health equity. However, enhancing innate cognitive ability through policy is challenging. Our argument does not imply narrow or unrealistic “cognitive enhancement” interventions. While educational interventions have been shown to improve IQ (Cahan & Cohen, 1989; Judd et al., 2022; Ritchie & Tucker-Drob, 2018), our study suggests that pre-existing cognitive ability's influence on health behaviors operates independently of formal education and socio-economic status.

Therefore, policy efforts may need to focus on modifying the mechanisms through which cognitive ability influences health behaviors. For risk-taking, peer characteristics are an important channel, but that is likely not addressed easily by policy. Rather, interventions promoting health literacy could be designed to compensate for lower cognitive ability by simplifying health information and tailoring prevention programs to different cognitive profiles. Such interventions, for instance implemented as school-based programs, would integrate health knowledge and decision-making skills into the curriculum and offer a scalable route for prevention. Such early interventions could help bridge gaps in health outcomes by ensuring that all individuals, regardless of cognitive ability, can process and act upon health information effectively. Finally, interventions that improve the contexts in which young people make choices (e.g., access to healthy food, safe spaces for activity, limiting exposure to harmful marketing) can buffer risks regardless of individual cognitive ability.

## CRediT authorship contribution statement

**Hendrik Jürges:** Writing – review & editing, Writing – original draft, Visualization, Methodology, Formal analysis, Data curation, Conceptualization. **Rasheda Khanam:** Writing – review & editing, Writing – original draft, Methodology, Data curation, Conceptualization.

## Ethical statement

This study used secondary analysis of de-identified LSAC data. LSAC is approved by the Australian Institute of Family Studies Human Research Ethics Committee. Parents provided written informed consent in the original data collection. According to LSAC guidelines, no separate ethics approval was required for use of these data.

## Declaration of generative AI and AI-assisted technologies in the writing process

During the preparation of this work the authors used ChatGPT 4o in order to improve the readability and language of the manuscript. After using this tool, the authors reviewed and edited the content as needed and take full responsibility for the content of the published article.

## Declaration of competing interest

The authors declare that they have no known competing financial interests or personal relationships that could have appeared to influence the work reported in this paper.

## Data Availability

The data used in this study are from the *Longitudinal Study of Australian Children (LSAC)*, which is conducted by the Australian Government Department of Social Services (DSS), in partnership with the Australian Institute of Family Studies (AIFS) and the Australian Bureau of Statistics (ABS). Access to LSAC data is restricted and subject to application and approval through the National Centre for Longitudinal Data (NCLD). Researchers can apply for access via the Australian Data Archive Dataverse: https://dataverse.ada.edu.au/dataverse/lsac. The authors do not have permission to share the data directly. Interested parties must obtain the data through the appropriate channels and meet all ethical and administrative requirements set by the data custodians.
