# Supplementary material for: Childhood IQ and adolescent health behavior
Source: SSM Popul Health. 2025 Nov 19;32:101887. doi: 10.1016/j.ssmph.2025.101887 (PMC12681829; doi:10.1016/j.ssmph.2025.101887)
Supplement: Multimedia component 1 [file mmc1.doc]

# Childhood IQ and Adolescent Health Behavior

Online Appendix

1. Tables and Figures
2. Variable description

# **A: Tables and Figures**

**Table A.1:** Sample description

|  | Unweighted | | | Weighted | | |  |  |
| --- | --- | --- | --- | --- | --- | --- | --- | --- |
| Label | Mean | | SD | Mean | | SD | Min | Max |
| Age in months (centered on 17 yrs) | -0.57 | | 4.33 | -0.3 | | 4.35 | -13 | 14 |
| Sex (boy = 1) | 0.5 | | 0.5 | 0.51 | | 0.5 | 0 | 1 |
|  |  | |  |  | |  |  |  |
| Mother’s region of birth: Oceania and antarctica | 0.68 | 0.47 | | 0.66 | 0.47 | | 0 | 1 |
| Mother’s region of birth: North-west Europe | 0.17 | 0.38 | | 0.17 | 0.38 | | 0 | 1 |
| Mother’s region of birth: Southern and eastern Europe | 0.04 | 0.2 | | 0.05 | 0.22 | | 0 | 1 |
| Mother’s region of birth: North Africa and the middle east | 0.01 | 0.08 | | 0.01 | 0.1 | | 0 | 1 |
| Mother’s region of birth: South-east Asia | 0.02 | 0.16 | | 0.03 | 0.17 | | 0 | 1 |
| Mother’s region of birth: North-east Asia | 0.02 | 0.14 | | 0.03 | 0.16 | | 0 | 1 |
| Mother’s region of birth: Southern and central Asia | 0.02 | 0.13 | | 0.02 | 0.13 | | 0 | 1 |
| Mother’s region of birth: Americas | 0.01 | 0.08 | | 0.01 | 0.08 | | 0 | 1 |
| Mother’s region of birth: Sub-Saharan Africa | 0.01 | 0.09 | | 0.01 | 0.09 | | 0 | 1 |
| Mother’s region of birth: Undisclosed | 0.02 | 0.14 | | 0.02 | 0.14 | | 0 | 1 |
|  | 0.62 | | 0.49 | 0.58 | | 0.49 | 0 | 1 |
| SDQ conduct problems | -0.05 | | 0.48 | -0.02 | | 0.49 | -0.62 | 1.85 |
| SDQ hyperactivity | -0.05 | | 0.5 | -0.02 | | 0.5 | -0.77 | 1.43 |
| SDQ emotional problems | -0.05 | | 0.47 | -0.03 | | 0.49 | -0.51 | 2.15 |
| SDQ peer problems | -0.07 | | 0.47 | -0.04 | | 0.48 | -0.55 | 2.35 |
|  |  | |  |  | |  |  |  |
| Number of older siblings | 0.55 | | 0.5 | 0.55 | | 0.5 | 0 | 1 |
| Number of hh members who smoke | 0.12 | | 0.41 | 0.16 | | 0.48 | 0 | 4 |
| Mother currently smokes | 0.17 | | 0.38 | 0.22 | | 0.41 | 0 | 1 |
| Mother currently drinks | 0.5 | | 0.69 | 0.46 | | 0.68 | 0 | 5.5 |
| Mother serves of vegetables/day | 2.22 | | 1.2 | 2.15 | | 1.21 | 0 | 6 |
| Mother serves of fruit/day | 1.56 | | 1.07 | 1.54 | | 1.1 | 0 | 6 |
| Father currently smokes | 0.17 | | 0.38 | 0.17 | | 0.38 | 0 | 1 |
| Father currently smokes NA | 0.17 | | 0.38 | 0.24 | | 0.42 | 0 | 1 |
| Father currently drinks | 1.13 | | 1.36 | 1.05 | | 1.3 | 0 | 12 |
| Father currently drinks NA | 0.16 | | 0.37 | 0.23 | | 0.42 | 0 | 1 |
| Out of home activities w child | 0.04 | | 0.49 | 0.01 | | 0.5 | -1.36 | 0.86 |
| Home activities w child | 0.04 | | 0.48 | 0.01 | | 0.49 | -1.59 | 1.19 |
|  |  | |  |  | |  |  |  |
| Number of children’s books | 37.76 | | 6.7 | 37.14 | | 7.7 | 0 | 40 |
| Mother’s age | 35.37 | | 4.81 | 34.85 | | 5 | 22 | 52 |
| HH rents home | 0.19 | | 0.4 | 0.26 | | 0.44 | 0 | 1 |
| HH financial rating | -0.07 | | 0.5 | -0.03 | | 0.5 | -1.35 | 1.7 |
| HH economic hardship | -0.07 | | 0.43 | -0.02 | | 0.48 | -0.35 | 2.36 |
| Father’s age | 37.8 | | 5.38 | 37.42 | | 5.32 | 19 | 69 |
| Father’s age NA | 0.09 | | 0.28 | 0.14 | | 0.34 | 0 | 1 |
| HH Socio-econ. position | 0.13 | | 0.49 | 0.03 | | 0.49 | -1.61 | 1.54 |
| Mother’s employment status: employed | 0.62 | | 0.49 | 0.58 | | 0.49 | 0 | 1 |
| Mother’s employment status: unemployed | 0.03 | | 0.17 | 0.04 | | 0.19 | 0 | 1 |
| Mother’s employment status: not in labor force | 0.35 | | 0.48 | 0.38 | | 0.49 | 0 | 1 |
| Parents’ highest educ. level: Less than year 12 | 0.05 | | 0.23 | 0.1 | | 0.29 | 0 | 1 |
| Parents’ highest educ. level: Year 12 | 0.1 | | 0.3 | 0.1 | | 0.3 | 0 | 1 |
| Parents’ highest educ. level: Certificate | 0.28 | | 0.45 | 0.32 | | 0.47 | 0 | 1 |
| Parents’ highest educ. level: Advanced diploma/diploma | 0.11 | | 0.32 | 0.11 | | 0.31 | 0 | 1 |
| Parents’ highest educ. level: Bachelor degree | 0.22 | | 0.41 | 0.18 | | 0.38 | 0 | 1 |
| Parents’ highest educ. level: Graduate diploma/certificate | 0.1 | | 0.3 | 0.09 | | 0.28 | 0 | 1 |
| Parents’ highest educ. level: Postgraduate degree | 0.13 | | 0.34 | 0.1 | | 0.31 | 0 | 1 |
| Mothers’ language proficiency mother tongue | 0.87 | | 0.34 | 0.84 | | 0.36 | 0 | 1 |
| Mothers’ language proficiency very well | 0.09 | | 0.29 | 0.1 | | 0.3 | 0 | 1 |
| Mothers’ language proficiency well | 0.03 | | 0.16 | 0.04 | | 0.2 | 0 | 1 |
| Mothers’ language proficiency not well/not at all | 0.01 | | 0.11 | 0.02 | | 0.14 | 0 | 1 |
|  |  | |  |  | |  |  |  |
| Remoteness: Major Cities | 0.65 | | 0.48 | 0.66 | | 0.47 | 0 | 1 |
| Remoteness: Inner Regional | 0.2 | | 0.4 | 0.2 | | 0.4 | 0 | 1 |
| Remoteness: Outer Regional | 0.13 | | 0.34 | 0.12 | | 0.32 | 0 | 1 |
| Remoteness: Remote Australia | 0.02 | | 0.14 | 0.02 | | 0.13 | 0 | 1 |
| Regional adv/disadvantage | 0.04 | | 0.51 | 0.01 | | 0.5 | -1.32 | 1.44 |
| School ICSEA | 0.06 | | 0.49 | 0.01 | | 0.49 | -2.01 | 1.2 |
| School grade 5 avg. NAPLAN score | 0.05 | | 0.49 | 0.01 | | 0.5 | -1.98 | 1.67 |
|  |  | |  |  | |  |  |  |
| Std. matrices | 0 | | 1 | -0.08 | | 1.02 | -3.58 | 3.17 |
|  |  | |  |  | |  |  |  |
| Risk-taking score | 0 | | 1 | 0.01 | | 1.04 | -1.14 | 4.97 |
| Health habit score | -0 | | 1 | 0.11 | | 1.03 | -1.34 | 3.51 |
|  |  | |  |  | |  |  |  |
| Longitudinal weight | 0.93 | | 0.47 | 1.17 | | 0.61 | 0.33 | 3.5 |

**Table A.2: Regression results**

|  | **Risk-taking score: Base** | **Risk-taking score: Full** | **Health habit score: Base** | **Health habit score: Full** |
| --- | --- | --- | --- | --- |
| (Intercept) | -0.032 (0.024) | -0.247 (0.210) | -0.023 (0.029) | -0.100 (0.249) |
| IQ (std. matrices) | -0.185 (0.043)*** | -0.107 (0.043)* | -0.428 (0.048)*** | -0.151 (0.044)*** |
| Age in months |  | -0.002 (0.007) |  | -0.004 (0.007) |
| Sex (boy = 1) |  | 0.079 (0.046)+ |  | 0.433 (0.047)*** |
| Age x Sex |  | -0.002 (0.010) |  | -0.010 (0.010) |
| Mother’s region of birth: North-west Europe |  | -0.050 (0.061) |  | -0.012 (0.060) |
| Mother’s region of birth: Southern and Eastern Europe |  | -0.366 (0.098)*** |  | -0.010 (0.153) |
| Mother’s region of birth: North Africa and the Middle East |  | -0.505 (0.194)** |  | 0.106 (0.219) |
| Mother’s region of birth: South-east Asia |  | -0.135 (0.133) |  | -0.171 (0.191) |
| Mother’s region of birth: North-east Asia |  | -0.315 (0.125)* |  | 0.055 (0.209) |
| Mother’s region of birth: Southern and central Asia |  | -0.161 (0.138) |  | 0.102 (0.174) |
| Mother’s region of birth: Americas |  | 0.271 (0.454) |  | 0.155 (0.217) |
| Mother’s region of birth: Sub-Saharan Africa |  | 0.110 (0.540) |  | -0.130 (0.219) |
| Mother’s region of birth: Undisclosed |  | -0.133 (0.130) |  | -0.234 (0.190) |
| SDQ: conduct problems |  | 0.077 (0.060) |  | 0.007 (0.051) |
| SDQ: hyperactivity |  | -0.009 (0.052) |  | 0.180 (0.046)*** |
| SDQ: emotional problems |  | -0.107 (0.053)* |  | 0.036 (0.046) |
| SDQ: peer problems |  | -0.017 (0.041) |  | 0.036 (0.053) |
| Older siblings in hh |  | 0.077 (0.045)+ |  | 0.138 (0.045)** |
| Number of hh members who smoke |  | 0.050 (0.083) |  | 0.156 (0.058)** |
| Mother currently smokes |  | 0.256 (0.071)*** |  | 0.165 (0.067)* |
| Mother currently drinks |  | 0.067 (0.034)* |  | 0.005 (0.030) |
| Mother serves of vegetables/day |  | 0.015 (0.019) |  | -0.017 (0.019) |
| Mother serves of fruit/day |  | -0.005 (0.018) |  | -0.050 (0.022)* |
| Father currently smokes |  | 0.006 (0.066) |  | 0.018 (0.067) |
| Father currently smokes NA |  | 0.063 (0.104) |  | 0.120 (0.095) |
| Father currently drinks |  | 0.040 (0.016)* |  | -0.006 (0.015) |
| Father currently drinks NA |  | 0.032 (0.101) |  | -0.008 (0.106) |
| Out of home activities w child |  | 0.094 (0.041)* |  | -0.053 (0.041) |
| Home activities w child |  | 0.085 (0.050)+ |  | -0.022 (0.045) |
| Number of children’s books |  | 0.002 (0.004) |  | -0.003 (0.004) |
| Parents’ highest educ. level: Year 12 |  | -0.078 (0.119) |  | -0.271 (0.102)** |
| Parents’ highest educ. level: Certificate |  | 0.003 (0.112) |  | -0.082 (0.095) |
| Parents’ highest educ. level: Advanced diploma/diploma |  | 0.101 (0.135) |  | -0.100 (0.103) |
| Parents’ highest educ. level: Bachelor’s degree |  | -0.028 (0.131) |  | -0.222 (0.115)+ |
| Parents’ highest educ. level: Graduate diploma/certificate |  | -0.163 (0.136) |  | -0.246 (0.119)* |
| Parents’ highest educ. level: Postgraduate degree |  | -0.063 (0.162) |  | -0.199 (0.137) |
| Mothers’ language proficiency: very well |  | 0.193 (0.094)* |  | 0.050 (0.122) |
| Mothers’ language proficiency: well |  | 0.114 (0.154) |  | 0.078 (0.190) |
| Mothers’ language proficiency: not well/not at all |  | 0.246 (0.139)+ |  | -0.352 (0.219) |
| Mother’s age |  | -0.007 (0.008) |  | -0.007 (0.006) |
| Mother’s employment status: unemployed |  | -0.014 (0.146) |  | 0.035 (0.128) |
| Mother’s employment status: not in labor force |  | -0.119 (0.048)* |  | 0.041 (0.046) |
| HH rents home |  | 0.081 (0.060) |  | 0.123 (0.056)* |
| HH financial rating |  | 0.049 (0.050) |  | -0.023 (0.048) |
| HH economic hardship |  | -0.009 (0.067) |  | 0.150 (0.059)* |
| Father’s age |  | 0.005 (0.005) |  | 0.008 (0.005) |
| Father’s age NA |  | 0.111 (0.128) |  | 0.017 (0.113) |
| HH Socio-econ. position |  | 0.039 (0.077) |  | -0.038 (0.077) |
| Regional adv/disadvantage |  | 0.007 (0.057) |  | -0.120 (0.054)* |
| School ICSEA |  | 0.099 (0.080) |  | -0.047 (0.076) |
| School grade 5 avg. NAPLAN score |  | -0.165 (0.077)* |  | -0.206 (0.073)** |
| Remoteness: Inner Regional |  | -0.105 (0.065) |  | -0.119 (0.060)* |
| Remoteness: Outer Regional |  | -0.033 (0.080) |  | 0.013 (0.065) |
| Remoteness: Remote Australia |  | 0.132 (0.154) |  | 0.019 (0.102) |
| Num.Obs. | 2253 | 2253 | 2253 | 2253 |
| R2 | 0.011 | 0.095 | 0.047 | 0.269 |
| R2 Adj. | 0.010 | 0.073 | 0.046 | 0.251 |
| AIC | 5745.5 | 5713.7 | 6129.9 | 5725.3 |
| BIC | 5757.0 | 6028.3 | 6141.4 | 6039.9 |
| RMSE | 0.87 | 0.84 | 0.94 | 0.84 |
| Std.Errors | by: pcodes | by: pcodes | by: pcodes | by: pcodes |

Notes: Cluster-robust standard errors in parentheses, + p<0.1; * p<0.05, ** p<0.01, *** p<0.001

*
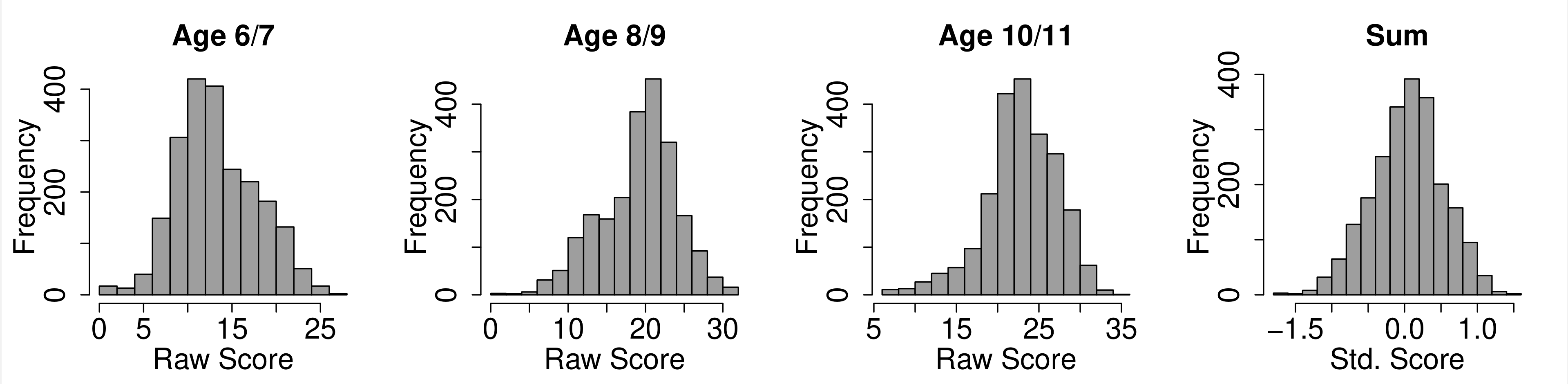
*

**Figure A.1**: Distribution of raw matrix reasoning scores at ages 6/7, 8/9, and 10/11, together with the standardized sum of the three raw scores. As the same (adaptive) test is administered at all ages, the distribution shifts to the right as children age, reflecting maturation.


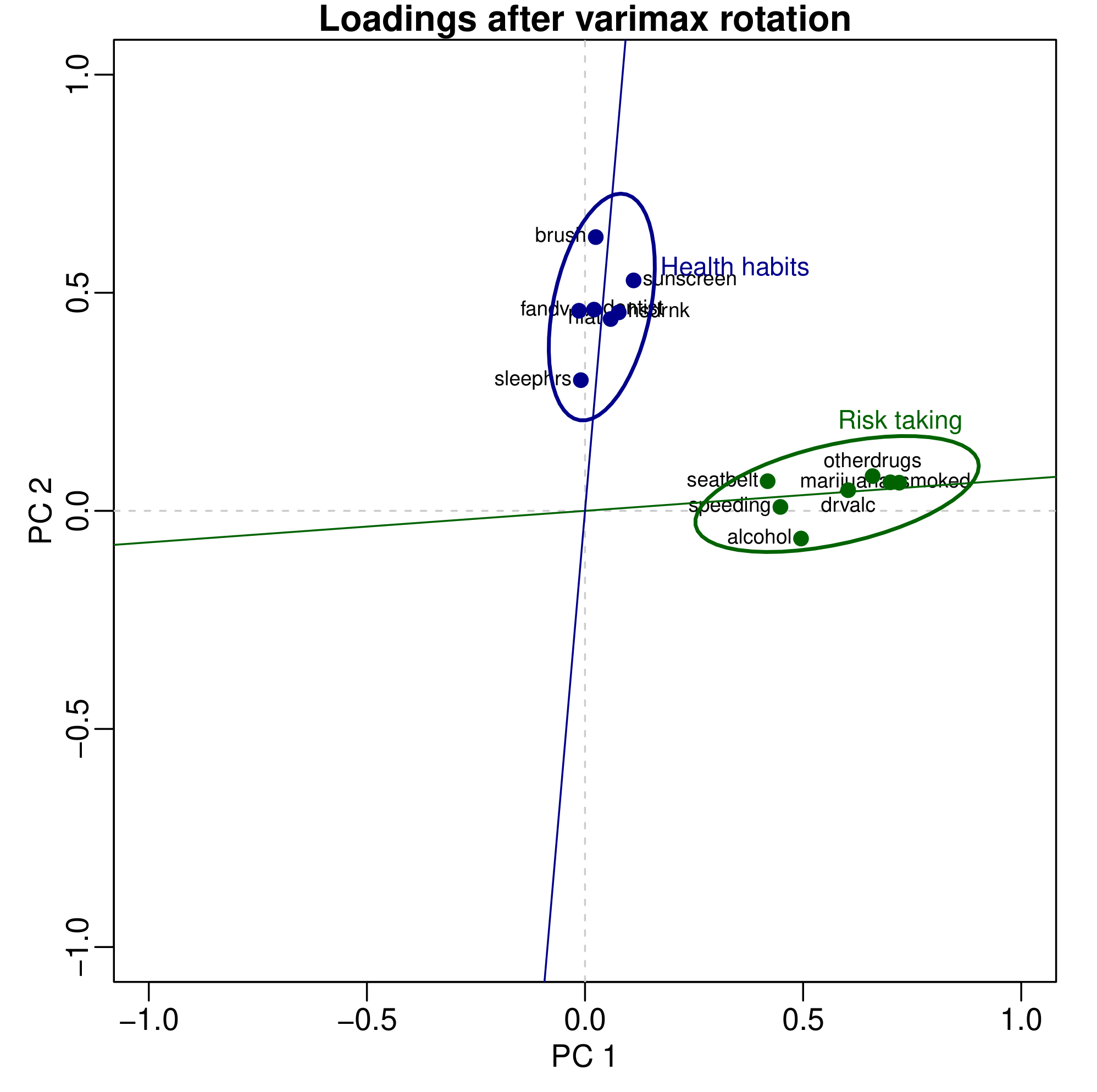


**Figure A.2:** Risk taking and health habits in the two-dimensional space after principal component analysis.


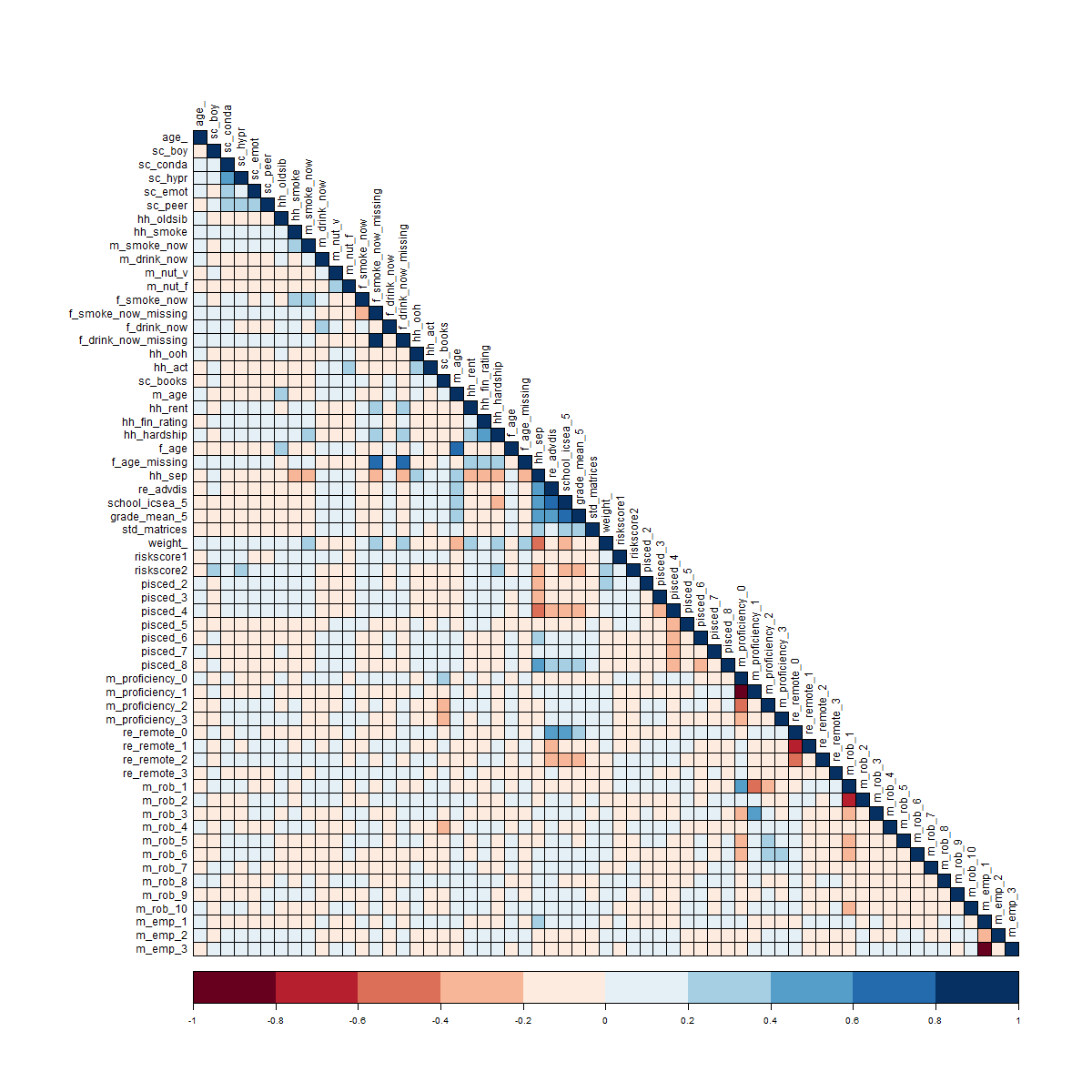


**Figure A.3:** Correlation plot (see next page for legend to short variable names)

Legend to Figure A2

| Short name | Variable label |
| --- | --- |
| age_ | Age in months (centered) |
| sc_boy | Sex (boy = 1) |
| sc_conda | SDQ: conduct problems |
| sc_hypr | SDQ: hyperactivity |
| sc_emot | SDQ: emotional problems |
| sc_peer | SDQ: peer problems |
| hh_oldsib | Number of older siblings |
| hh_smoke | Number of hh members who smoke |
| m_smoke_now | Mother currently smokes |
| m_drink_now | Mother currently drinks |
| m_nut_v | Mother serves of vegetables/day |
| m_nut_f | Mother serves of fruit/day |
| f_smoke_now | Father currently smokes |
| f_smoke_now_missing | Father currently smokes NA |
| f_drink_now | Father currently drinks |
| f_drink_now_missing | Father currently drinks NA |
| hh_ooh | Out of home activities w child |
| hh_act | Home activities w child |
| sc_books | Number of children’s books |
| m_age | Mother’s age |
| hh_rent | HH rents home |
| hh_fin_rating | HH financial rating |
| hh_hardship | HH economic hardship |
| f_age | Father’s age |
| f_age_missing | Father’s age NA |
| hh_sep | HH Socio-econ. position |
| re_advdis | Regional adv/disadvantage |
| school_icsea_5 | School ICSEA |
| grade_mean_5 | School grade 5 avg. NAPLAN score |
| std_matrices | Std. matrices |
| weight_ | Longitudinal weight |
| riskscore1 | Risk-taking score |
| riskscore2 | Health habit score |
| pisced_2 | Parents’ highest educ. level: Less than year 12 |
| pisced_3 | Parents’ highest educ. level: Year 12 |
| pisced_4 | Parents’ highest educ. level: Certificate |
| pisced_5 | Parents’ highest educ. level: Advanced diploma/diploma |
| pisced_6 | Parents’ highest educ. level: Bachelor degree |
| pisced_7 | Parents’ highest educ. level: Graduate diploma/certificate |
| pisced_8 | Parents’ highest educ. level: Postgraduate degree |
| m_proficiency_0 | Mothers’ language proficiency mother tongue |
| m_proficiency_1 | Mothers’ language proficiency very well |
| m_proficiency_2 | Mothers’ language proficiency well |
| m_proficiency_3 | Mothers’ language proficiency not well/not at all |
| re_remote_0 | Remoteness: Major Cities |
| re_remote_1 | Remoteness: Inner Regional |
| re_remote_2 | Remoteness: Outer Regional |
| re_remote_3 | Remoteness: Remote Australia |
| m_rob_1 | Mother’s region of birth: OCEANIA AND ANTARCTICA |
| m_rob_2 | Mother’s region of birth: NORTH-WEST EUROPE |
| m_rob_3 | Mother’s region of birth: SOUTHERN AND EASTERN EUROPE |
| m_rob_4 | Mother’s region of birth: NORTH AFRICA AND THE MIDDLE EAST |
| m_rob_5 | Mother’s region of birth: SOUTH-EAST ASIA |
| m_rob_6 | Mother’s region of birth: NORTH-EAST ASIA |
| m_rob_7 | Mother’s region of birth: SOUTHERN AND CENTRAL ASIA |
| m_rob_8 | Mother’s region of birth: AMERICAS |
| m_rob_9 | Mother’s region of birth: SUB-SAHARAN AFRICA |
| m_rob_10 | Mother’s region of birth: UNDISCLOSED |
| m_emp_1 | Mother’s employment status: employed |
| m_emp_2 | Mother’s employment status: unemployed |
| m_emp_3 | Mother’s employment status: not in labor force |

# **B: Variable description**

## B.1: Health behavior measures

**Use of legal and illegal substances**:

- “Number of cigarettes you had during the last 7 days, including yesterday?”
- “Number of alcoholic drinks you had during the last seven days, including yesterday?”
- “Have you *ever* tried marijuana” Yes or No
- “Have you *ever* tried sniffing/had medicines for non-medical purposes/tried other drugs” Yes or No

**Driving too fast or without helmet or seatbelt**:

“Try to remember *the last 10 time*s you drove a car or other vehicle. On how many occasions have you done any of the following?

- drove more than 25 km/h over the limit
- did not wear a seat belt (or helmet if riding motorbike) at all/for part of the trip

During the last 12 months:

- did you drive a car or other vehicle while under the influence of alcohol or drugs or have you been a passenger in a car or other vehicle when the driver was under the influence of alcohol or drugs? Yes or No

**Dental care and prevention:**

Yesterday, how often did you brush your teeth? (number of times (0 to 3))

When did you last see a dentist? (number of years ago)

**Sleep**

1. About what time do you fall asleep on a usual school or work night?
2. About what time do you wake up in the morning on a usual school or work day?

Sleep duration is difference between (B) and (A): less than 7 hrs to more than 9 hrs = 1, 7 to 9 hrs = 0

**Sunscreen**:

“Think about what you do when you spend time outside during the summer on a warm sunny day. How often do you wear sunscreen (of at least SPF 15+)?”

0 Never versus 1 Rarely; 2 Sometimes; 3 Often; 4 Always

**Nutrition**:

“Thinking about *yesterday*, how often did you have X?

0=Not at all, 1=once, 2=twice, 3=more than twice.”

- High fat food: (Sum of the following > 2)
  - a meat pie, hamburger, hot dog, sausage or sausage roll
  - hot chips or French fries
  - potato chips or savoury snacks such as ‘Twisties’
  - biscuits, doughnuts, cake or chocolate
- High sugar drinks: (Sum of the following > 1=1, else 0)
  - fruit juice
  - soft drink or cordial, not diet or sugar free soft drink or cordial
- Fruit and Vegetables: (Sum of the following<3 = 1, else =0 )
  - fresh fruit
  - cooked vegetables
  - raw vegetables or salad

## B.2: Peer characteristics measured at age 14/15

| **Item** | **Wording** | **Dimension** |
| --- | --- | --- |
| A | They read books for fun. | Positive |
| B | They try to get away with things | Moral |
| C | I get into trouble when I am with them | Moral |
| D | They work hard at school | Positive |
| E | They get into a lot of trouble at school | Moral |
| F | They do well in school | Positive |
| G | They are good at sports | --- |
| H | They are mean to other kids | Moral |
| I | They cheat on tests | Moral |
| J | They go to church or religious services | Moral |
| K | They dislike school | Positive (reversed) |
| L | They are respectful of teachers | Positive |
| M | They think being popular is more important than getting good grades | Positive (reversed) |
| N | They are involved in lots of activities outside of school (e.g. lessons or sports) | --- |
| O | They make me do things I am ashamed of | Moral |
| P | They get into lots of fights | Risk taking |
| Q | They smoke cigarettes | Risk taking |
| R | They drink alcohol | Risk taking |
| S | They have broken the law (e.g., shoplifts, vandalism, steals cars) | Risk taking |
| T | They try drugs (e.g., marijuana, ecstasy) | Risk taking |

Note: Question text: “This set of questions is about the kids that you spend time with. You might know these kids from school, your neighbourhood, or anywhere else. Think about these kids when you answer each of the questions.” Answer categories: 1 None of them; 2 One or two of them; 3 Some of them; 4 Most of them; 5 All of them.
